# Benefits of Neuropsychiatric Phenomics: Example of the 5-Lipoxygenase-Leptin-Alzheimer Connection

**DOI:** 10.1155/2010/838164

**Published:** 2010-06-27

**Authors:** Hari Manev, Radmila Manev

**Affiliations:** Department of Psychiatry, University of Illinois at Chicago, Chicago, IL 60612, USA

## Abstract

Phenomics is a systematic study of phenotypes on a genomewide scale that is expected to unravel, as of yet, unsuspected functional roles of the genome. It remains to be determined how to optimally approach and analyze the available phenomics databases to spearhead innovation in neuropsychiatry. By serendipitously connecting two unrelated phenotypes of increased blood levels of the adipokine leptin, a molecule that regulates appetite, in 5-lipoxygenase- (5-LOX) deficient mice and patients with a lower risk for Alzheimer's disease (AD), we postulated a leptin-mediated basis for beneficial effects of *ALOX5* (a gene encoding 5-LOX) gene-deficiency in AD. We suggest that it might be possible to avoid relying on serendipity and develop data-mining tools capable of extracting from phenomics databases indications for such novel hypotheses. Hence, we provide an example of using a free-access *Arrowsmith* two-node search interface to identify *ALOX5* as unsuspected putative mechanisms for the previously described clinical association between increased plasma levels of leptin and a lower risk of incident dementia and AD.

A narrow definition of “*phenomics*” is “a systematic study of phenotypes on a genome-wide scale” [1]. Phenomics has emerged as natural result of recent successes in high-throughput genotyping. It is expected that phenomics research would unravel, as of yet, unsuspected functional roles of the genome. Notwithstanding some promise in genetic linkage studies in the fields of neurology and psychiatry, it has become a truism that neuropsychiatric disorders are too complex to be caused by a single gene disturbance. 

Although new phenotypes are continuously being discovered that can be associated with neuropsychiatric disorders, it remains to be determined how to optimally approach and analyze the available phenomics databases to spearhead innovation in neuropsychiatry. Case in point is the recent report of a curious association between increased plasma levels of the adipokine leptin, a molecule that regulates appetite, and a lower risk of incident dementia and Alzheimer disease (AD) [2]. Although a number of genes have been associated with AD, no obvious genetic marker could account for the observed negative association between increased plasma leptin and AD. Finding such a marker would open novel lines of AD research.

With availability of cross-species genotype/phenotype resources such as *PhenomicDB* (http://www.phenomicdb.de/) one could benefit from comparisons of human phenotypes with those of experimental animal models. An obvious question arises—is there a mouse gene knockout that leads to increased plasma leptin levels and decreased AD-like pathology? It would be helpful if one could address this question in an interactive free-access phenomics database such as *PhenomicDB*. However, using this database we were unable to obtain a simple answer to the above hypothetical question. As it often happens, serendipity pointed us into the right direction. Through our previous research interest into links between the inflammatory enzyme 5-lipoxygenase (5-LOX) and AD [3], we were aware of a mouse knockout model in which *ALOX5* gene, which encodes 5-LOX, had been made deficient. When this 5-LOX deficiency was transferred into a transgenic mouse model of AD, the Tg2576, the amyloid-*β* deposition in the brains of Tg2576 mice lacking 5-LOX, was reduced by 64%–80% compared with Tg2576 controls [4]. Prompted by the findings of the AD-protective phenotype of increased plasma leptin [2], we recalled that this leptin phenotype was recently described in the *ALOX5* mouse knockout [5]. Thus, the same *ALOX5* knockout that caused a reduced AD-like pathology in mice [4] also increased their plasma levels of leptin [5]. 

It might be possible to avoid relying on serendipity and develop data-mining tools capable of extracting from phenomics databases answers to questions similar to the one we have posed in this article. For example, one could apply a tool such as the *Arrowsmith* two-node search interface (http://arrowsmith.psych.uic.edu/arrowsmith_uic/index.html). This tool has been developed to search articles in MEDLINE [6]. Briefly, one inputs two separate PubMed queries, “A” and “C”. Any articles present in both A and C *are removed* so that the analysis will consider only *indirect* linkages between the two sets. Then the software identifies all words and 2- and 3-word phrases found in the titles of the articles in both A and C. These so-called B-terms are processed and ranked according to the predicted probability that they will be relevant in pointing to a meaningful link across the two literatures. The user can further streamline the B-list, for example, by selecting only the gene/protein names.

Hence, we used the free-access *Arrowsmith* interface to test whether the *ALOX5* gene could be discovered as a new link between elevated plasma leptin levels and AD by a PubMed search. We had chosen the advanced mode and entered “increased plasma leptin” in search “A”. This generated 2094 items. We entered “Alzheimer” in search “C”. This generated 53659 items. Then, the software generated the “B” list with 5030 items. We filtered this list using the option “semantics” by selecting “gene or protein names”. This reduced the list to 359 items. To further reduce this number, in the next step, we selected a filter termed “frequency” and changed the default settings from “fewer” to “more”, leaving the rest unchanged. This reduced the list to 41 items. First on the list of genes/proteins was “ampk” (adenosine monophosphate-activated protein kinase), and second was “alox5” (other high-ranking genes/proteins were peptide yy, Chop, matrix metalloproteinase MMP-2, phospholipid transfer protein, and serum-glucocorticoid-inducible-kinase 1). Asking the software to identify literature associated with “alox5” returned only two publications. One of them was the paper on elevated plasma leptin in *ALOX5* knockout mice [5]. Thus, it appears that in this case we would have been able to discover the 5-LOX-leptin-AD link without prior knowledge about the leptin phenotype of *ALOX5* knockout mice. It has to be stressed that a simple use of PubMed, that is, by entering together the three terms “leptin alox5 alzheimer” or “leptin 5-lipoxygenase Alzheimer” did not generate any hits.

With this commentary, we have illustrated how phenomics databases and data-mining interfaces can be used to find unsuspected links such as the example of a putative link between 5-LOX, leptin, and AD. Follow-up research studies are needed to understand the exact nature of such links. For example, to explore whether 5-LOX deficiency could benefit AD patients not only via the previously suggested mechanism that involves gamma secretase [4] but also in part due to 5-LOX-mediated alterations of circulating leptin levels. Recent study has identified a role for 5-LOX polymorphism in AD in a population from Northern Italy [7]. Based on similarities between mouse and human phenotypes described in this article, it appears that including leptin and 5-LOX in future clinical studies with this and similar AD patient populations is warranted.

## References

[1] Bilder RM, Sabb FW, Cannon TD (2009). Phenomics: the systematic study of phenotypes on a genome-wide scale. *Neuroscience*.

[2] Lieb W, Beiser AS, Vasan RS (2009). Association of plasma leptin levels with incident Alzheimer disease and MRI measures of brain aging. *Journal of the American Medical Association*.

[3] Ikonomovic MD, Abrahamson EE, Uz T, Manev H, DeKosky ST (2008). Increased 5-lipoxygenase immunoreactivity in the hippocampus of patients with Alzheimer’s disease. *Journal of Histochemistry and Cytochemistry*.

[4] Firuzi O, Zhuo J, Chinnici CM, Wisniewski T, Praticò D (2008). 5-Lipoxygenase gene disruption reduces amyloid-*β* pathology in a mouse model of Alzheimer's disease. *FASEB Journal*.

[5] Mehrabian M, Schulthess FT, Nebohacova M (2008). Identification of *ALOX5* as a gene regulating adiposity and pancreatic function. *Diabetologia*.

[6] Smalheiser NR, Torvik VI, Zhou W (2009). Arrowsmith two-node search interface: a tutorial on finding meaningful links between two disparate sets of articles in MEDLINE. *Computer Methods and Programs in Biomedicine*.

[7] Listì F, Caruso C, Lio D (2010). Role of cyclooxygenase-2 and 5-lipoxygenase polymorphisms in Alzheimer’s disease in a population from Northern Italy: implication for pharmacogenomics. *Journal of Alzheimer's Disease*.

